# Pharmacological Characterization of the Mechanisms Involved in Delayed Calcium Deregulation in SH-SY5Y Cells Challenged with Methadone

**DOI:** 10.1155/2012/642482

**Published:** 2012-06-17

**Authors:** Sergio Perez-Alvarez, Maria E. Solesio, Maria D. Cuenca-Lopez, Raquel M. Melero-Fernández de Mera, Carlos Villalobos, Hanna Kmita, Maria F. Galindo, Joaquin Jordán

**Affiliations:** ^1^Neuropharmacology, Department of Medical Sciences, School of Medicine, University of Castilla-La Mancha (UCLM), 02006 Albacete, Spain; ^2^Translational Neuropsychopharmacology Unit, Albacete University Hospital Center, Albacete, Spain; ^3^Institute of Molecular Biology and Genetics (IBGM), C/ Sanz y Forés 3, 47003 Valladolid, Spain; ^4^Laboratory of Bioenergetics, Institute of Molecular Biology and Biotechnology, Faculty of Biology, Adam Mickiewicz University, Umultowska 89, 61-614 Poznan, Poland; ^5^Instituto de Investigación en Discapacidades Neurológicas, UCLM, Albacete, Spain

## Abstract

Previously, we have shown that SH-SY5Y cells exposed to high concentrations of methadone died due to a necrotic-like cell death mechanism related to delayed calcium deregulation (DCD). In this study, we show that, in terms of their Ca^2+^ responses to 0.5 mM methadone, SH-SY5Y cells can be pooled into four different groups. In a broad pharmacological survey, the relevance of different Ca^2+^-related mechanisms on methadone-induced DCD was investigated including extracellular calcium, L-type Ca^2+^ channels, *μ*-opioid receptor, mitochondrial inner membrane potential, mitochondrial ATP synthesis, mitochondrial Ca^2+^/2Na^+^-exchanger, reactive oxygen species, and mitochondrial permeability transition. Only those compounds targeting mitochondria such as oligomycin, FCCP, CGP 37157, and cyclosporine A were able to amend methadone-induced Ca^2+^ dyshomeostasis suggesting that methadone induces DCD by modulating the ability of mitochondria to handle Ca^2+^. Consistently, mitochondria became dramatically shorter and rounder in the presence of methadone. Furthermore, analysis of oxygen uptake by isolated rat liver mitochondria suggested that methadone affected mitochondrial Ca^2+^ uptake in a respiratory substrate-dependent way. We conclude that methadone causes failure of intracellular Ca^2+^ homeostasis, and this effect is associated with morphological and functional changes of mitochondria. Likely, this mechanism contributes to degenerative side effects associated with methadone treatment.

## 1. Introduction

Methadone (D,L-methadone hydrochloride) is frequently used in different therapies including opioid addiction [1], long-lasting analgesics in cancer and neuropathic pain syndromes [1–3]. However, numerous reports indicate a negative impact on human cognition by chronic exposure to opioid drugs. Patients subjected to methadone maintenance programs show impaired cognitive abilities in aspects such as psychomotor performance, information processing, attention, problem solving, memory, decision making, reaction time, and emotional facial expression recognition [4–10].

Changes in the cytosolic free-calcium concentration ([Ca^2+^]_cyt_) are involved in control of a large number of cellular and physiological processes including neuronal excitability, synaptic plasticity, and gene transcription [11, 12]. However, the physiological Ca^2+^ signal can switch to a death signal when the [Ca^2+^]_cyt_  increases dramatically. For example, excitotoxic high glutamate concentrations result in an initial transient increase in [Ca^2+^]_cyt  _that is followed by a delayed, irreversible rise in [Ca^2+^]_cyt_  known as delayed calcium deregulation  (DCD). Although several steps preceding DCD remain to be clarified, there is evidence that DCD is the irreversible end point of a sequence involving mitochondrial Ca^2+^ overloading. DCD precedes and reliably predicts the necrotic death of cultured neurons [13].

Mitochondria are important for cellular Ca^2+^  homeostasis. They buffer variations in Ca^2+^ concentrations by taking up Ca^2+^ when and where [Ca^2+^]_cyt_ levels are passing a threshold level above which the mitochondrial Ca^2+^ uniporter is activated, and slowly release Ca^2+^ back to the cytosol when [Ca^2+^]_cyt_ drop below this point [14]. Mitochondrial Ca^2+^ overload, if large and sustained enough, may contribute to mitochondria permeability transition pore (MPTP) formation and ultimately lead to cell death [11, 15]. Because, mitochondria may accumulate a considerable amount of Ca^2+^ during neurotoxic exposure, a possibility is that DCD may represent the final consequence of mitochondrial Ca^2+^ overload. MPTP is a large, proteinaceous, Ca^2+^-activated, proton- and ADP-inhibited voltage-dependent pore. It spans the inner and outer mitochondrial membrane allowing the passage of ions and substrates less than 1.5 kDa. Characteristically, opening of the MPTP is inhibited by cyclosporin A [16, 17].

SH-SY5Y cells are considered a suitable model for investigating opioid-mediated responses in neurons. These cells express both *μ*- and *δ*-opioid receptors [18]. In previous studies, we showed that SH-SY5Y cells exposed to high concentrations of methadone (0.5 mM) died through a necrotic-like cell death mechanism and that methadone may induce changes in the [Ca^2+^]_cyt_ [19, 20]. However, the underlying mechanisms causing alterations of the [Ca^2+^]_cyt_ in SH-SY5Y cells in the presence of methadone remained unknown. Therefore, the aim of the present study was to investigate those mechanisms. A clear understanding of the factors that mediate this phenomenon might help to resolve the mechanisms that promote neuronal cell death during methadone-induced cognitive damage.

## 2. Material and Methods

### 2.1. Cells Cultures

 SH-SY5Y cells (ATCC) were plated at a density of 5.3 × 10^4^ cells/cm^2^ on *μ*-Dish 35 mm High IbiTreat (ibidi GmbH, Martinsried, München, Germany) as previously reported [21].

### 2.2. Calcium Measurements

 Changes in [Ca^2+^]_cyt_ in SH-SY5Y cells were measured by loading cells with the calcium probe Fura2/AM and using an inverted fluorescence microscope (Nikon Eclipse TE2000-S) as described elsewhere [22]. Cells were perfused with a medium containing 140 mM NaCl, 5 mM KCl, 1 mM MgCl_2_, 2.5 mM CaCl_2_, 10 mM Hepes, and 11 mM Glucose, pH 7.35. Ratios of fluorescence emission excited at 340 and 380 nm were captured every 5 seconds [22]. Methadone or other compounds were added from 1000x stock solutions to reach the appropriate final concentrations. The effects of selected compounds were tested on [Ca^2+^]_cyt_ in the absence and the presence of 0.5 mM methadone in different sets of cells. Cells were pooled according to the dynamics of the rises in [Ca^2+^]. 

### 2.3. Mitochondrial Morphology 

To directly visualize mitochondrial morphology changes in intact cells overexpressing pDsRed2-mito plasmid (Clontech Laboratories, Inc. Mountain View, CA, USA) a Leica SP2 confocal microscope (63 × 1.4 NA objective) was used. For transfection of cells the reagent Lipofectamine was used (Invitrogen, Carlsbad, CA, USA) [23].

### 2.4. Mitochondria Isolation

 Rat liver mitochondria were isolated in MSH/EDTA and MSH media containing 210 mM mannitol, 70 mM sucrose, 5 mM Hepes, with or without 1 mM EDTA, pH 7.4, by differential centrifugation according to the standard procedure [24]. Mitochondrial protein concentration was measured using the Micro BCA Protein Reagent Kit. The mitochondrial suspensions were kept on ice and immediately used for measurements of oxygen-uptake rate. 

### 2.5. Mitochondrial Oxygen Uptake

The rate of oxygen uptake of isolated rat liver mitochondria was measured at 37°C in a water-thermostatized incubation chamber with a computer-controlled Clark-type O_2_ electrode (Oxygraph, Hansatech, UK) in 0.5 ml incubation buffer (145 mM KCl, 30 mM Hepes, 5 mM KH_2_PO_4_, 3 mM MgCl_2_, 0.1 mM EGTA, 0.1% defatted BSA, pH 7.4). The respiratory substrates used were complex I- or complex II-linked, 2.5 mM glutamate/2.5 mM malate or 5 mM succinate in the presence of 2 *μ*M rotenone. The following additions were applied: 250 *μ*M ADP, 200 *μ*M CaCl_2_, 0.4 *μ*M FCCP, and 0.5 mM methadone. For estimating mitochondrial Ca^2+^ uptake in isolated mitochondria a Ca^2+^ index was calculated, which denotes the ratio of oxygen-uptake rate triggered by addition of 200 *μ*M Ca^2+^ to previous oxygen uptake rate.

## 3. Results and Discussion

**Figure 1:**
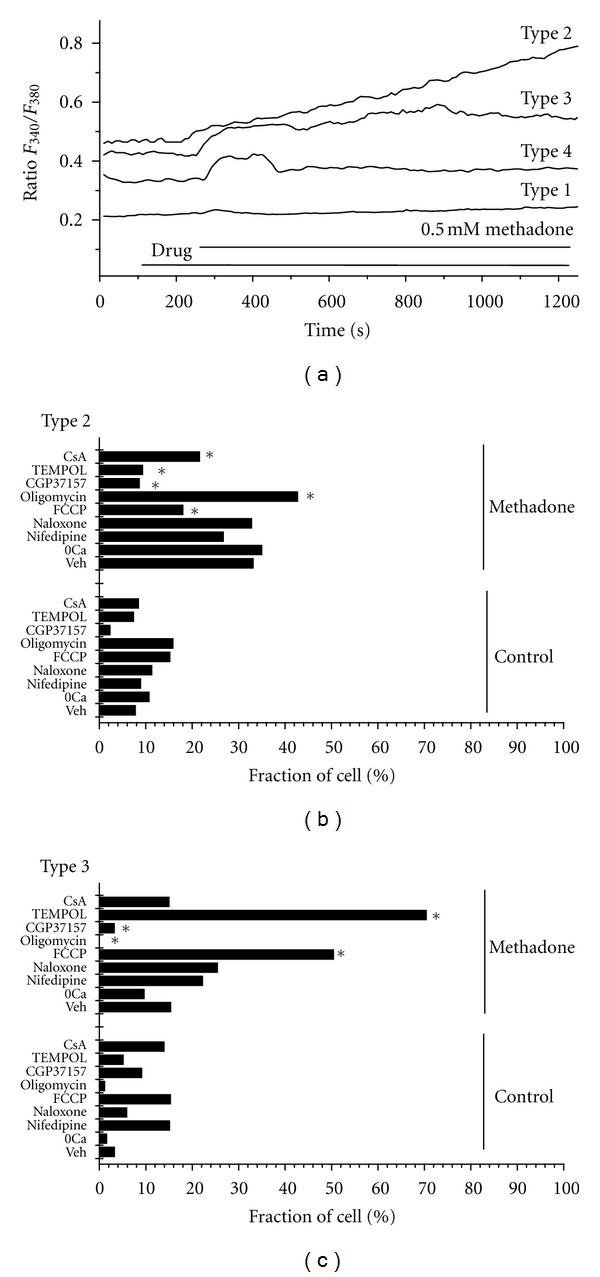
Methadone induces Ca^2+^ dyshomeostasis. (a). Effects of 0.5 mM methadone on [Ca^2+^]_cyt_ in SH-SY5Y cells. Representative recordings of the four different types of response including no [Ca^2+^]_cyt_ increase (type 1), early [Ca^2+^]_cyt_ sustained increase (type 2), delayed [Ca^2+^]_cyt_ increase (type 3), and transient [Ca^2+^]_cyt_ increase (Type 4). (b)-(c)Relative abundance (%) of SH-SY5Y cells that display type 2 (b) or type 3 (c) recordings without (Control) and with methadone treatment (Veh). Also shown are the effects of several treatments on the above mentioned relative abundance. Treatments include removal of extracellular calcium (0 Ca), L-type Ca^2+^ channel blocker Nifedipine (2 *μ*M); opioid receptor antagonist Naloxone (50 *μ*M), mitochondrial uncoupler FCCP (1 *μ*M); ATP synthase inhibitor oligomycin (10 *μ*g/ml), mitochondria Na^+^/Ca^2+^ exchanger CGP37157 (25 *μ*M); superoxide dismutase mimetic TEMPOL (0.2 *μ*M) or MPTP antagonist cyclosporine A (1 *μ*M, CsA). All treatments were performed 5 min prior to addition of 0.5 mM methadone. Data represent results obtained in at least 3 independent experiments. *P < 0.05; Student's *t*-test versus basal conditions, (Veh).

In this study, we have investigated the mechanisms involved in methadone-induced rises in [Ca^2+^]_cyt_ in SH-SY5Y cells. Consistent with previous observations from our laboratory [19] and other data [25], methadone induced a rise in [Ca^2+^]_cyt_ in most of the SH-SY5Y cells. However, the effect of methadone differed considerably across cells. Analysis of the dynamics of the [Ca^2+^]_cyt_ recordings in the absence and the presence of methadone suggested that four different types of calcium recordings can be observed in SH-SY5Y cells.  Figure 1(a) shows typical examples of recordings. For the first group of cells no rise in [Ca^2+^]_cyt_ was observed during the entire period (30 min) of measurements (type 1). A second group of cells was unable to regulate [Ca^2+^]_cyt_ homeostasis shortly after the addition of methadone (type 2) and [Ca^2+^]_cyt_ increased continuously. A third group displayed [Ca^2+^]_cyt_  deregulation with a delay of 15–20 minutes (type 3). Finally, the fourth group was able to control [Ca^2+^]_cyt_ after an initial increase (type 4). Groups 1 and 4 represent, respectively, nonresponsive of cells and cells responding with transient rise in [Ca^2+^]_cyt_ and amount 20.82 and 31.38% of the cells. However, types 2 and 3 displayed short or delayed continuous rise in [Ca^2+^]_cyt_ that may be considered as early or delayed Ca^2+^ deregulation according to the literature [26–29]. The abundance of cells showing deregulation (type 2 and type 3) amount to 32.83 and 14.92%, of the total, respectively.

We have analyzed the relative abundance of the four types of recordings described above in the absence (Control) and the presence (Methadone) of 0.5 mM methadone. The results obtained are shown in Figures 1(b) and 1(c). Clearly, methadone decreased type 4 (transient responsive cells) and increased types 2 and 3 (deregulated cells). These results suggest that methadone may induce a short or delayed Ca^2+^ deregulation in SH-SY5Y cells. 

The mechanisms underlying the observed quantitative changes in the types of Ca^2+^ responses mediated by methadone are unknown. Therefore, we performed a comprehensive pharmacological survey to study the possible contribution of different Ca^2+  ^related mechanism to the response to methadone. As illustrated in Figures 1(b) and 1(c) and discussed below, a 5 min pretreatment of SH-SY5Y cells with different conditions and drugs affected the methadone-induced [Ca^2+^]_cyt_ response, monitored as changed frequencies of the different types of the responses. We found that in the absence of extracellular calcium (0 Ca) no change in responses to methadone was observed (*n* = 291 cells). These results suggest that Ca^2+^ entry does not contribute to the reported changes in [Ca^2+^]_cyt_. To confirm that extracellular Ca^2+^  was not involved in these responses we tested the effect of methadone in the presence of nifedipine, a specific isopropyl L-type Ca^2+^ channel blocker. Nifedipine (2 *μ*M) did not modify the effects of methadone on the [Ca^2+^]_cyt_ in SH-SY5Y cells (*n* = 226 cells). So, we ruled out the involvement of this voltage-dependent channel family in methadone-induced [Ca^2+^]_cyt_ variations. We asked then whether intracellular Ca^2+^ stores could contribute to the responses to methadone. Cells were treated with thapsigargin (1–100 *μ*M). To deplete intracellular Ca^2+^ stores before methadone treatment. As expected, thapsigargin induced a significant release in Ca^2+^ from the endoplasmic reticulum, causing a transient increase in the [Ca^2+^]_i_ that failed to returned to the basal level within a 10-minute period. A detailed observation of the methadone induced rise in [Ca^2+^]_i_ shows that cell responses were partially affected by this treatment, suggesting that the rise in calcium is partially due to release from thapsigargin-sensitive, intracellular Ca^2+^ stores (*n* = 146 cells). We found that in untreated cells addition of thapsigargin induced a transient increase in [Ca^2+^] that has been attributed to a leakage of Ca^2+^ from the endoplasmic reticulum. This effect of thapsigargin alone interferes the interpretation of the type 2 and 3 responses after methadone addition (data not shown).

Another possible mechanism is the activation of endogenous opioid receptors. To test this possibility, we treated the cells with 50 *μ*M Naloxone), the competitive antagonist of the *μ*-opioid receptor. We found that this treatment did not modify the relative abundance of any of the four types of cell populations (*n* = 80 cells, 4 exp). This result suggests that the changes in [Ca^2+^]_cyt_ and DCD induced by methadone in SH-SY5Y cells are independent of opioid receptors. In agreement with the lack of *μ*-opioid receptor participation as found here, it has been reported that methadone-toxic pathways are not mediated by *μ* receptors [19, 30–32].

 To test the possible contribution of mitochondria we used the mitochondrial uncoupler carbonyl cyanide p-trifluoromethoxyphenylhydrazone (FCCP; 1 *μ*M) Ca^2+^ uptake by mitochondria depends strongly on mitochondrial potential (*ΔΨm*). It is well established that FCCP collapses mitochondrial potential and abolishes the ability of mitochondria to take up Ca^2+^. We found that in untreated cells, addition of FCCP induced a transient increase in [Ca^2+^] that has been attributed to leakage of Ca^2+^ from depolarized mitochondria. We found that FCCP induced a 44% decrease in the appearance of type 2 cells whereas increased the relative abundance of cells showing a type 3 response by 2.7-fold in FCCP (Figures 1(b)-1(c)). FCCP abolished the presence of type 1 cells.

The above results suggest that mitochondria are likely involved in the response to methadone. Nevertheless, it must be taken into account that as the simple usage of protonophores does not allow a clear-cut study of the role of mitochondrial Ca^2+^ transport as they also may cause a lowering of the ATP/ADP ratio, thereby affecting ATP-dependent Ca^2+^ pumps [14]. An approach that has been previously exploited to investigate the role of mitochondria in synaptosomal Ca^2+^ homeostasis involves inhibition of mitochondrial ATP synthesis by oligomycin and application of glycolysis as the source of ATP and independent manipulation of *ΔΨ m* with specific respiratory chain inhibitors [33, 34]. Inhibition of ATP synthase by oligomycin prevents mitochondrial oxidative phosphorylation, but unlike protonophore addition, it does not cause hydrolysis of cytoplasmically generated ATP. It has been reported that DCD might result from a failure in Ca^2+^ extrusion caused by cytoplasmic ATP depletion [26]. Therefore, we tested the effects of a short, 5 min, incubation with 10 *μ*g/ml oligomycin on Ca^2+^ responses. Under these conditions, oligomycin alone did not modify the [Ca^2+^]_cyt_ responses during the 30 min recording period. However, oligomycin did alter the [Ca^2+^]_cyt_ responses to methadone. Specifically, the abundance of SH-SY5Y cells showing a type 2 response were increased whereas the pool of cells showing a DCD-related type 3 response were lost. Additionally, oligomycin induced a 3.3-fold increase in cells showing a type 4 response. To test further the contribution of mitochondria we investigated the possible role of the mitochondrial Ca^2+^/2Na^+^ exchanger. Mitochondrial Ca^2+^ efflux is normally primarily regulated by a Ca^2+^/2Na^+^ exchanger. To block mitochondrial Ca^2+^ exit we used CGP37157 (50 *μ*M), an inhibitor of the Ca^2+^/2Na^+^-exchanger. A five min exposure of cells to CGP37157 significantly increased the proportion of cells showing no rise in [Ca^2+^]_cyt_ in response to methadone during the entire [Ca^2+^]_cyt_ measurement period (type 1) (data not shown) because CGP37157 resulted in a drastic decrease in the population of cells showing either type 2 (74%) or type 3 (85%) responses. In addition, the relative abundance of cells showing a type 1 response returned to the value obtained in untreated cells. These results support data suggesting the relevance of mitochondria in methadone-induced DCD (Figures 1(b)-1(c)).

To test contribution of reactive oxygen species we used the cell-permeable, small molecule compound TEMPOL (4-hydroxy-2,2,6,6-tetramethylpiperidinyloxy) to mimic superoxide dismutase activity. In the presence of TEMPOL (0.2 *μ*M; *n* = 80 cells), cells responded to methadone in a different way. Specifically, TEMPOL decreased the number of cells showing a type 2 response by ~70%, whereas the abundance of cells showing a type 3 response was largely increased (3.5 fold). Types 1 and 4 were nearly not present. Consistent with our results, Nicholls et al. [28] suggested that enhanced ROS is a consequence rather than a cause of DCD. In their studies, they applied a novel technique to monitor the bioenergetic status of in situ mitochondria in cultured neurons in a model of glutamate excitotoxicity. In agreement with this, a general ineffectiveness of antioxidants to decrease DCD in the presence of glutamate has been observed [35]. Finally, we tested the contribution of the mitochondrial permeability transition (MPTP). Additional efflux of mitochondrial Ca^2+^ can occur by induction of MPTP formation, which is dependent on the mitochondrial matrix Ca^2+^ concentration and can be inhibited by cyclosporine A [16, 17]. To evaluate MPTP participation we administered CsA (1 *μ*M). CsA diminished the occurrence of response type 2 by 36% (*n* = 89 cells) in cell cultures challenged with 0.5 mM methadone. Moreover, consistent with the hypothesized role of the pore in DCD, CsA induced a 2-fold increase in cells showing type 4 response. However, interpretation of these results is difficult because CsA may also inhibit the mitochondrial Ca^2+^ uniporter in some instances.

Taken together, our data indicate that only drugs affecting mitochondrial handling of Ca^2+^, such as oligomycin, FCCP, CGP 37157, and cyclosporine A, were able to modulate methadone-induced delayed calcium deregulation in SH-SY5Y cells. We therefore conclude that methadone-induced dyshomeostasis is caused by improper functioning of mechanisms that directly control mitochondrial activity rather than a participation of plasma membrane Ca^2+^ channels or opioid receptors. 

**Figure 2:**
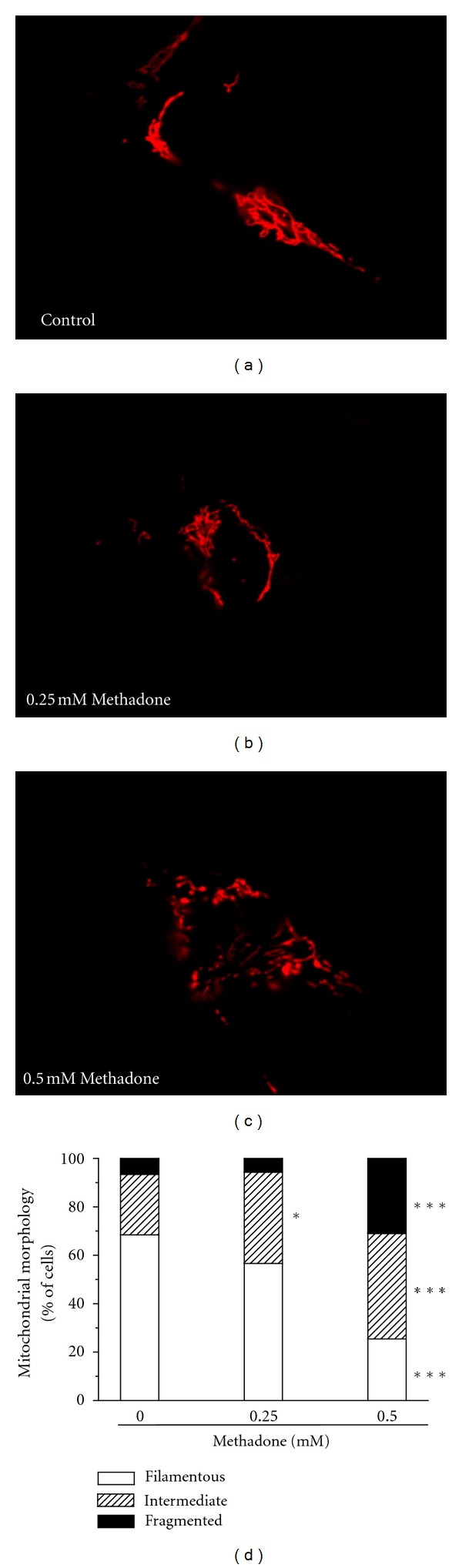
Methadone alters mitochondrial morphology. Morphology of methadone-treated cells was studied by confocal imaging of SH-SY5Y cells transfected with the pDsRed2-mito vector. Twenty-four hours after transfection, cell cultures were incubated for 3 h in the absence or presence of 0.25 or 0.5 mM methadone. Shown are images of the representative mitochondrial morphology in non-treated cells (control, (a)) or cells treated with methadone ((b), 0.25 mM; (c), 0.5 mM). Scale bar indicates 10 *μ*m. (d), The fractions of cells with filamentous, intermediate or punctuate mitochondrial patterns were determined in at least 6 independent cultures. (*P < 0.05; ***P < 0.001; Student's *t*-test versus basal conditions as indicated.)

Next, the effect of methadone on mitochondrial morphology was studied in SH-SY5Y cells transfected with pDsRed2-mito. In untreated cultures, mitochondria presented a long and tubular morphology (Figure 2(a)), which became dramatically shorter and rounder upon three hours of methadone treatment (Figures 2(b)–2(d)). Cell counting of the different mitochondrial morphologies (filamentous, mixed and fragmented) indicated that methadone, in a dose-dependent manner, induced mitochondrial fragmentation (Figure 2(d)). This effect seems contradictory to our earlier observations that methadone failed to induce mitochondrial swelling in isolated rat liver mitochondria [19]. Possibly, the fragmentation effect of methadone on mitochondria is mediated by calcium. In fact, our data support this hypothesis, and indicate a role of DCD in methadone-induced toxicity. In agreement with this, it has been reported that the MPTP opens under pseudopathological conditions with relatively high Ca^2+^ and low ATP concentrations [15] as was the case in our previous experiments with SH-SY5Y cells [19]. The rupture of the mitochondrial membrane caused by Ca^2+^ overload reduces the number of “healthy” mitochondria and this will affect crucial neuronal functions including synaptic transmission and axonal transport.

**Figure 3:**
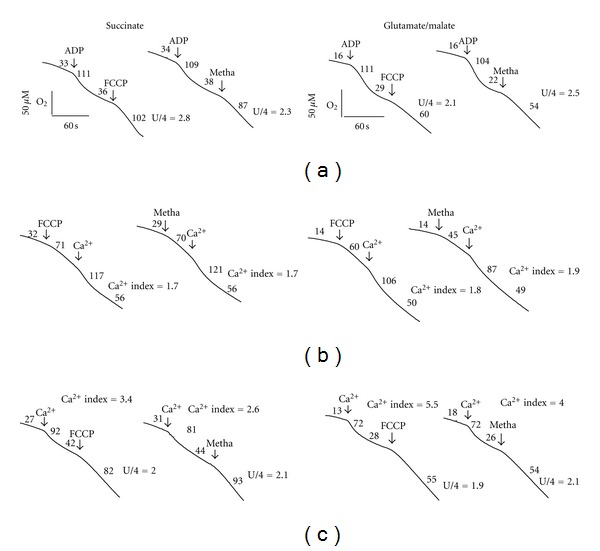
Methadone affects mitochondrial Ca^2+^ uptake in a respiratory substrate-dependent way. Oxygen uptake by isolated liver mitochondria was determined using a Clark electrode in the presence of 5 mM succinate or 2.5 mM glutamate/2.5 mM malate as substrates. (a) Uncoupling capacity of FCCP and methadone. (b) The effect of FCCP and methadone on Ca^2+^ uptake. (c) The effect of Ca^2+^ uptake on the uncoupling capacity of FCCP and methadone. Additions: 250 *μ*M ADP, 200 *μ*M CaCl_2_, 0.4 *μ*M FCCP, and 0.5 mM methadone. Traces shown are the means obtained from four independent mitochondria preparations. Numbers on the traces indicate respiration rates at 37°C in nmol oxygen·mg protein^−1^·min^−1^.

Finally, the effect of methadone on mitochondrial Ca^2+^ uptake was studied (Figure 3). We used a Clark electrode and applied different respiratory substrates, namely succinate and glutamate/malate. The respiratory chain is less dependent on the presence of ΔΨm for succinate than for glutamate/malate. Methadone is known to cause uncoupling [19]. Therefore, as a control, 0.4 *μ*M FCCP was used because this concentration of FCCP resulted in an increase of oxygen-uptake rate comparable with the uptake rate calculated for 0.5 mM methadone (Figure 3(a)). The calculated values of the state U (uncoupled state) to state 4 (resting state) ratios (U/4 ratios) were as follows: for succinate 2.8 ± 0.5 (+FCCP) and 2.3 ± 0.4 (+methadone), and for glutamate/malate 2.1 ± 0.5 (+FCCP) and 2.5 ± 0.4 (+methadone). Then, we checked the effect of FCCP and methadone on Ca^2+^ uptake by mitochondria (Figures 3(b) and 3(c)). For this purpose a Ca^2+^ *index *was calculated, which denotes the ratio of oxygen-uptake rate triggered by addition of 200 *μ*M Ca^2+^ to previous oxygen uptake rate. The values of the Ca^2+^ index were as follows: for succinate 3.0 ± 0.7 (control), 1.7 ± 0.3 (+FCCP), and 1.7 ± 0.4 (+methadone), and for glutamate/malate 4.8 ± 0.3 (control), 1.8 ± 0.4 (+FCCP), and 1.9 ± 0.5 (+methadone). Thus, the effects of FCCP and methadone on Ca^2+^ uptake are comparable, although in the presence of glutamate/malate the effect appears to be much more pronounced. This probably results from a stronger uncoupling effect of methadone on glutamate/malate access to the respiratory chain and a consecutive additional impairment of Ca^2+^ uptake. Therefore, the effect of methadone on Ca^2+^ uptake by mitochondria may be dependent on the respiratory substrates. On the other hand, when the values of the U/4 ratios calculated in the absence and presence of Ca^2+^ uptake were compared (Figures 3(a) and 3(c)), a distinctive decrease was observed in traces recorded in the presence of Ca^2+^ uptake (Figure 3(c)). The calculated values of U/4 ratio decreased as follows: for succinate from 2.8 ± 0.5 to 2.0 ± 0.2 (+FCCP) and 2.3 ± 0.4 to 2.1 ± 0.3 (+methadone) and for glutamate/malate 2.1 ± 0.5 to 1.9 ± 0.3 (+FCCP) and 2.5 ± 0.4 to 2.1 ± 0.2 (+methadone). This could be caused by the reduction of a ΔΨ component of the protomotive force as a result of Ca^2+^ uptake, leading to a decrease of uncoupling capacity by FCCP and methadone. 

The data presented indicate that methadone induces DCD in SH-SY5Y cells by altering the capacity of mitochondria to handle calcium, and correlates with distinct changes of mitochondrial morphology. These morphological changes, in turn, can be associated with mitochondrial damage and cell death. Interestingly, swollen mitochondria have been observed in the context of neurodegenerative diseases [36, 37]. An imbalance in mitochondrial Ca^2+^ homeostasis might be important for both early and late stages of the observed side effects and, perhaps account for some of the observed clinical symptoms, for example, memory impairment.

## Abbreviations

FCCP: Carbonylcyanide-4-(trifluoromethoxy)-phenylhydrazone
[Ca^2+^]_cyt_: Cytosolic Ca^2+^ concentrations
DCD: Delayed calcium deregulation
ΔΨm: Mitochondrial inner membrane potential
MPTP: Mitochondrial permeability transition pore
ROS: Reactive oxygen species.

## References

[1] Krantz MJ, Mehler PS (2004). Treating opioid dependence. Growing implications for primary care. *Archives of Internal Medicine*.

[2] Mercadante S (2007). Opioid titration in cancer pain: a critical review. *European Journal of Pain*.

[3] Mercadante S (1997). Methadone in cancer pain. *European Journal of Pain*.

[4] Gritz ER, Shiffman SM, Jarvik ME (1975). Physiological and psychological effects of methadone in man. *Archives of General Psychiatry*.

[5] Darke S, Sims J, McDonald S, Wickes W (2000). Cognitive impairment among methadone maintenance patients. *Addiction*.

[6] Specka M, Finkbeiner T, Lodemann E, Leifert K, Kluwig J, Gastpar M (2000). Cognitive-motor performance of methadone-maintained patients. *European Addiction Research*.

[7] Curran HV, Kleckham J, Bearn J, Strang J, Wanigaratne S (2001). Effects of methadone on cognition, mood and craving in detoxifying opiate addicts: a dose-response study. *Psychopharmacology*.

[8] Kornreich C, Foisy ML, Philippot P (2003). Impaired emotional facial expression recognition in alcoholics, opiate dependence subjects, methadone maintained subjects and mixed alcohol-opiate antecedents subjects compared with normal controls. *Psychiatry Research*.

[9] Verdejo A, Toribio I, Orozco C, Puente KL, Pérez-García M (2005). Neuropsychological functioning in methadone maintenance patients versus abstinent heroin abusers. *Drug and Alcohol Dependence*.

[10] Mintzer MZ, Stitzer ML (2002). Cognitive impairment in methadone maintenance patients. *Drug and Alcohol Dependence*.

[11] Berridge MJ (1998). Neuronal calcium signaling. *Neuron*.

[12] Berridge MJ, Bootman MD, Lipp P (1998). Calcium—a life and death signal. *Nature*.

[13] Tymianski M, Charlton MP, Carlen PL, Tator CH (1993). Source specificity of early calcium neurotoxicity in cultured embryonic spinal neurons. *Journal of Neuroscience*.

[14] Budd SL, Nicholls DG (1996). Mitochondria, calcium regulation, and acute glutamate excitotoxicity in cultured cerebellar granule cells. *Journal of Neurochemistry*.

[15] Bernardi P, Forte M (2007). The mitochondrial permeability transition pore. *Novartis Foundation Symposium*.

[16] Bernardi P (1992). Modulation of the mitochondrial cyclosporin A-sensitive permeability transition pore by the proton electrochemical gradient. Evidence that the pore can be opened by membrane depolarization. *The Journal of Biological Chemistry*.

[17] Zoratti M, Szabo I (1995). The mitochondrial permeability transition. *Biochimica et Biophysica Acta*.

[18] Kazmi SMI, Mishra RK (1987). Comparative pharmacological properties and functional coupling of *μ* and *δ* opioid receptor sites in human neuroblastoma SH-SY5Y cells. *Molecular Pharmacology*.

[19] Perez-Alvarez S, Cuenca-Lopez MD, de Mera RMMF (2010). Methadone induces necrotic-like cell death in SH-SY5Y cells by an impairment of mitochondrial ATP synthesis. *Biochimica et Biophysica Acta*.

[20] Perez-Alvarez S, Iglesias-Guimarais V, Solesio ME (2011). Methadone induces CAD degradation and AIF-mediated necrotic-like cell death in neuroblastoma cells. *Pharmacological Research*.

[21] Perez-Alvarez S, Solesio ME, Manzanares J, Jordán J, Galindo MF (2009). Lactacystin requires reactive oxygen species and Bax redistribution to induce mitochondria-mediated cell death. *British Journal of Pharmacology*.

[22] Garcia-Martinez EM, Sanz-Blasco S, Karachitos A (2010). Mitochondria and calcium flux as targets of neuroprotection caused by minocycline in cerebellar granule cells. *Biochemical Pharmacology*.

[23] Gomez-Lazaro M, Bonekamp NA, Galindo MF, Jordán J, Schrader M (2008). 6-Hydroxydopamine (6-OHDA) induces Drp1-dependent mitochondrial fragmentation in SH-SY5Y cells. *Free Radical Biology and Medicine*.

[24] Jordán J, Galindo MF, Tornero D (2002). Superoxide anions mediate veratridine-induced cytochrome c release and caspase activity in bovine chromaffin cells. *British Journal of Pharmacology*.

[25] Pakkanen JS, Nousiainen H, Yli-Kauhaluoma J (2005). Methadone increases intracellular calcium in SH-SY5Y and SH-EP1-h*α*7 cells by activating neuronal nicotinic acetylcholine receptors. *Journal of Neurochemistry*.

[26] Nicholls DG, Budd SL (2000). Mitochondria and neuronal survival. *Physiological Reviews*.

[27] Nicholls DG, Chalmers S (2004). The integration of mitochondrial calcium transport and storage. *Journal of Bioenergetics and Biomembranes*.

[28] Nicholls DG, Johnson-Cadwell L, Vesce S, Jekabsons M, Yadava N (2007). Bioenergetics of mitochondria in cultured neurons and their role in glutamate excitotoxicity. *Journal of Neuroscience Research*.

[29] Randall RD, Thayer SA (1992). Glutamate-induced calcium transient triggers delayed calcium overload and neurotoxicity in rat hippocampal neurons. *Journal of Neuroscience*.

[30] Zagon IS, McLaughlin PJ (2003). Opioids and the apoptotic pathway in human cancer cells. *Neuropeptides*.

[31] Ren XH, Zhao J, Pu L, Ling K, Yin DL, Pei G (1998). Differential neurotoxicity of etorphine-like opiates: lack of correlation with their ability to activate opiate receptors. *Toxicon*.

[32] Yin DL, Ren XH, Zheng ZL (1997). Etorphine inhibits cell growth and induces apoptosis in SK-N-SH cells: involvement of pertussis toxin-sensitive G proteins. *Neuroscience Research*.

[33] Akerman KEO, Nicholls DG (1981). Intrasynaptosomal compartmentation of calcium during depolarization-induced calcium uptake across the plasma membrane. *Biochimica et Biophysica Acta*.

[34] Scott ID, Nicholls DG (1980). Energy transduction in intact synaptosomes: influence of plasma-membrane depolarization on the respiration and membrane potential of internal mitochondria determined in situ. *Biochemical Journal*.

[35] Vergun O, Sobolevsky AI, Yelshansky MV, Keelan J, Khodorov BI, Duchen MR (2001). Exploration of the role of reactive oxygen species in glutamate neurotoxicity in rat hippocampal neurones in culture. *Journal of Physiology*.

[36] Ferreirinha F, Quattrini A, Pirozzi M (2004). Axonal degeneration in paraplegin-deficient mice is associated with abnormal mitochondria and impairment of axonal transport. *The Journal of Clinical Investigation*.

[37] Menzies FM, Cookson MR, Taylor RW (2002). Mitochondrial dysfunction in a cell culture model of familial amyotrophic lateral sclerosis. *Brain*.

